# EHMTI-0397. Significance of the economy productivity losses assessment in cause of migraine in whole population of Russia

**DOI:** 10.1186/1129-2377-15-S1-J5

**Published:** 2014-09-18

**Authors:** I Fokin

**Affiliations:** 1 Neurology, 64 Moscow Municipal Clinic, Moscow, Russia

## Objective

Migraine is very important disease because of middle age (25 -55 years) working people disability. Migraine patients cannot work, have temporal disability< that can be reason of the large social and economy losses. In social aspect migraine leads to disadaptation in patient’s life and decrease its quality.

## Methods

We used Russian State Standard and Statistical Committee materials for January 2013 and own own questionnaires studies of migraine patients.

## Results

The production of gross revenue in 2012 was 10 863.4 billion rubles. There are 14 543 migraine patients (17%) in general working group. In calculation of general number of working population in Russia for 2012 (87.9 millions) and coefficient the value of production works 0.75, approximately evaluation of the part of internal gross revenue of each worker in calculation in US dollars is compose 2910 dollars per year. Taking account the numbers of working population in Russia and coefficient of taking part in the valuation of making production 0.75, the general quantity of migraine patients day lost is 6 working days per year increasing 0,7, that will be 395,55 millions patients-days or 1.58 millions in year. General gross revenue lost in US dollars will be 4604.2 millions. Mean cost of 1 day of disability is 90 rubles. Mean disability losses per year is constitute of 31 412 880 rubles.

## Conclusion

Migraine is high social-economical significance disease and its adequate therapy can decrease social and economical expenses of the population and increase the quality of life in migraine patients.

No conflict of interest.

